# A heart full of nails: complex management of multiple nail gun cardiac injuries

**DOI:** 10.1093/ehjcr/ytaf168

**Published:** 2025-04-08

**Authors:** Shaun Abid, Anton Stolear, Stuart Zarich

**Affiliations:** Department of Internal Medicine, Yale New Haven Health—Bridgeport Hospital, 267 Grant Street, Bridgeport, CT 06610, USA; Department of Cardiology, Yale New Haven Health—Bridgeport Hospital, 267 Grant Street, Bridgeport, CT 06610, USA; Department of Cardiology, Yale New Haven Health—Bridgeport Hospital, 267 Grant Street, Bridgeport, CT 06610, USA

**Keywords:** Penetrating cardiac trauma, Intracardiac foreign bodies, Emergency sternotomy, Cardiac tamponade, Multidisciplinary management, Valve replacement surgery, Case report

A 54-year-old male presented after a suicide attempt, suffering severe cardiac and extracardiac injuries from firing a nail gun into both temples (*Figure 1A*) and his chest. Imaging showed six 5 cm nails embedded in his heart: two in the left ventricle, two in the right ventricle, one traversing the ascending aorta into the left atrium, and one entering the right ventricle from the left parasternal chest wall (*Figure 1B–D*). These injuries resulted in haemorrhagic pericardial effusion with tamponade, acute aortic regurgitation, mitral valve/pulmonary artery perforations, and a ventricular septal defect.

**Figure 1:**
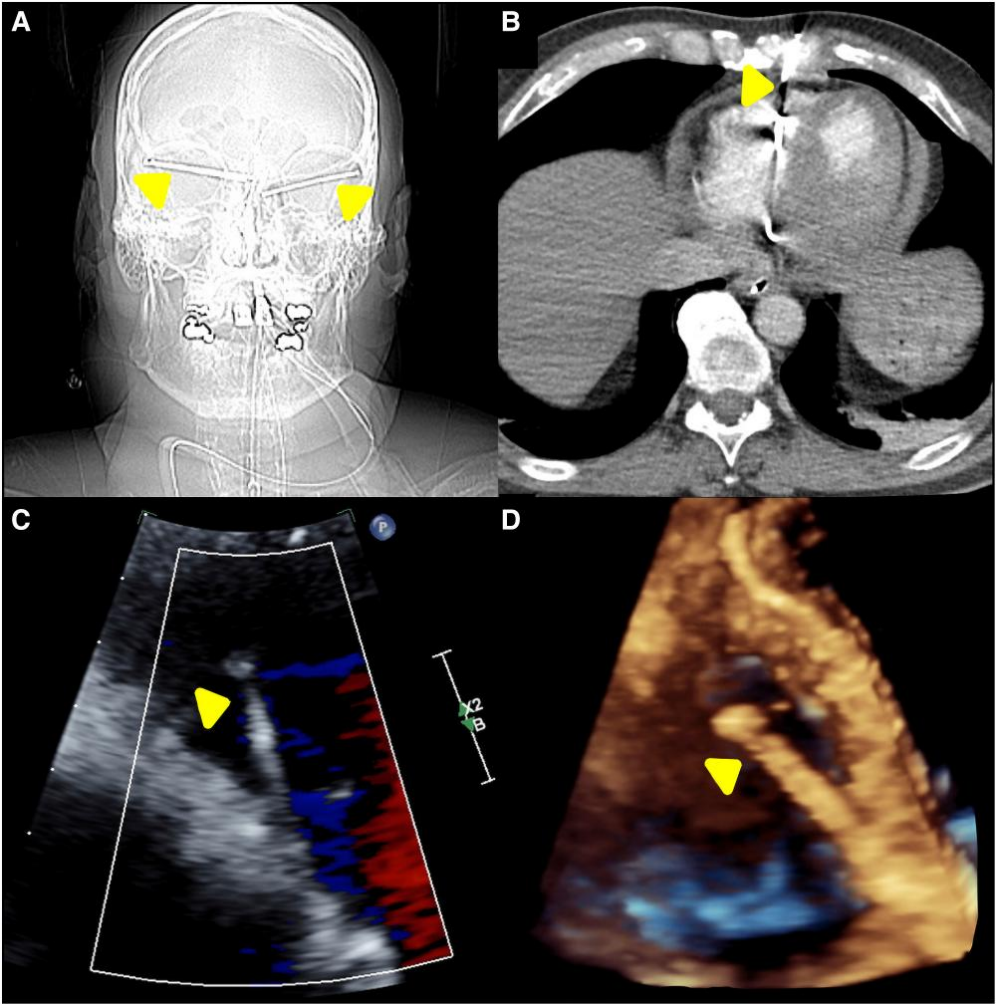
*(A)* Radiographic image of multiple nails traversing the bilateral orbits and penetrating intracranially. Two nails are seen entering through the right temple, with one terminating in the medial right orbit. Another nail enters through the left temple and terminates in the medial left orbit (arrows). *(B)* Chest CT demonstrating a single nail penetrating the right ventricle. The nail is seen traversing the chest wall, breaching the pericardium, and embedding within the right ventricular cavity (arrow). *(C)* Two-dimensional transthoracic echocardiogram showing a nail piercing the right ventricle. The nail penetrates the right ventricular free wall, with its head protruding into the ventricular cavity (arrow). *(D)* Three-dimensional transthoracic echocardiogram depicting a nail lodged within the interventricular septum. The nail remains embedded within the septum, with its head visible within the ventricular cavity (arrow).

Neurosurgical intervention was deemed secondary to the cardiac injuries. An emergency sternotomy was performed, evacuating 300 cc of blood from the pericardium and removing five intracardiac nails, while the sixth nail in the right ventricle was deemed too dangerous to remove due to the risk of significant vascular injury. Aortic valve replacement with a 27 mm Edwards bioprosthetic valve was required due to extensive damage to the non-coronary and left coronary cusps. Injuries to the septum, pulmonary artery, left atrium, anterior leaflet of the mitral valve, and left ventricle were repaired. Neurosurgery removed two nails from the right side of the head and one from the left. The patient required subsequent pulmonary valve replacement with a 29 mm Edwards Inspiris Resilia valve and mitral valve repair with a 36 mm annuloplasty band. Due to diffuse chest wall bleeding, re-exploration was required, during which bleeding was successfully controlled. Over the ensuing days, both cardiac and neurological status improved, and a psychiatric evaluation was initiated to address the underlying mental health concerns. At his 3-month follow-up, he was doing well, with a stable repeat echocardiogram.

This case highlights the challenges of managing cardiac trauma involving multi-chamber, large vessel, and valvular injuries, along with haemodynamic compromise. Prompt surgical intervention and multidisciplinary care are essential.

**Consent:** The patient's consent has been obtained for publication.

**Funding:** The authors report no specific funding related to this article.

## Data Availability

No data was generated or analysed for or in support of this article.

